# The SIPHER Consortium: Introducing the new UK hub for systems science in public health and health economic research

**DOI:** 10.12688/wellcomeopenres.15534.1

**Published:** 2019-11-12

**Authors:** Petra Meier, Robin Purshouse, Marion Bain, Clare Bambra, Richard Bentall, Mark Birkin, John Brazier, Alan Brennan, Mark Bryan, Julian Cox, Greg Fell, Elizabeth Goyder, Alison Heppenstall, John Holmes, Ceri Hughes, Asif Ishaq, Visakan Kadirkamanathan, Nik Lomax, Ruth Lupton, Suzy Paisley, Katherine Smith, Ellen Stewart, Mark Strong, Elizabeth Such, Aki Tsuchiya, Craig Watkins

**Affiliations:** 1 School of Health and Related Research, University of Sheffield, Sheffield, S1 4DA, UK; 2 Department of Automatic Control & Systems Engineering, University of Sheffield, Sheffield, S1 3JD, UK; 3 Population Health Directorate, Scottish Government, Edinburgh, EH1 3DG, UK; 4 Institute of Health and Society, Newcastle University, Newcastle upon Tyne, NE1 4LP, UK; 5 Clinical Psychology Unit, Department of Psychology, University of Sheffield, Sheffield, S1 2LT, UK; 6 Leeds Institute for Data Analytics, University of Leeds, Leeds, LS2 9NL, UK; 7 Department of Economics, University of Sheffield, Sheffield, S1 4DT, UK; 8 Greater Manchester Combined Authority, Manchester, M1 6EU, UK; 9 Sheffield City Council, Sheffield, S1 2HH, UK; 10 Inclusive Growth Analysis Unit, University of Manchester, Manchester, M13 9PL, UK; 11 School of Social Work & Social Policy, University of Strathclyde, Glasgow, G4 0LT, UK; 12 Centre for Biomedicine, Self & Society, University of Edinburgh, Edinburgh, EH8 9AG, UK; 13 Department of Urban Studies and Planning, University of Sheffield, Sheffield, S1 4DP, UK

**Keywords:** Health in All Policies, Wellbeing, Non-Communicable Disease, Prevention, Inequalities, Economic evaluation, Complex systems, Public Policy, Inclusive Growth, Housing, Public Mental Health, Adverse Childhood Experiences

## Abstract

The conditions in which we are born, grow, live, work and age are key drivers of health and inequalities in life chances. To maximise health and wellbeing across the whole population, we need well-coordinated action across government sectors, in areas including economic, education, welfare, labour market and housing policy. Current research struggles to offer effective decision support on the cross-sector strategic alignment of policies, and to generate evidence that gives budget holders the confidence to change the way major investment decisions are made. This open letter introduces a new research initiative in this space. The SIPHER ( Systems Science in Public Health and Health Economics Research) Consortium brings together a multi-disciplinary group of scientists from across six universities, three government partners at local, regional and national level, and ten practice partner organisations. The Consortium’s vision is a shift from health policy to healthy public policy, where the wellbeing impacts of policies are a core consideration across government sectors. Researchers and policy makers will jointly tackle fundamental questions about: a) the complex causal relationships between upstream policies and wellbeing, economic and equality outcomes; b) the multi-sectoral appraisal of costs and benefits of alternative investment options; c) public values and preferences for different outcomes, and how necessary trade-offs can be negotiated; and d) creating the conditions for intelligence-led adaptive policy design that maximises progress against economic, social and health goals. Whilst our methods will be adaptable across policy topics and jurisdictions, we will initially focus on four policy areas: Inclusive Economic Growth, Adverse Childhood Experiences, Mental Wellbeing and Housing.

## Background

### Framing healthy public policy as a complex systems problem

Non-communicable diseases (NCDs) account for 89% of UK premature deaths at an estimated societal cost of at least £100bn a year ^1^. Health policy often tries to prevent NCDs and reduce health inequalities by tackling health behaviours (e.g. alcohol and tobacco consumption, exercise, diet). Yet social and structural determinants of health (SDoH) account for a far greater burden of NCD and contribute more to health inequalities than individual behaviours ^2^. Thus, the greatest prevention opportunities lie outside the health sector’s control but within reach of sectors such as welfare, housing, and employment ^3^. Three main challenges hinder efforts to realise these opportunities. First, non-health policymakers often lack incentives, resources, expertise and evidence to assess their policies’ health impacts. Second, policy costs and benefits are spread across sectors, making it harder to identify synergies, negotiate trade-offs and mobilise cross-sector political will. Third, each policy area is an ‘open’ and dynamic system, subject to external forces (e.g. the global economy) that can shift rapidly, causing policies to become untenable, ineffective or to have unintended consequences.

The move towards healthy public policy (sometimes known as ‘Health in All Policies’ or ‘Wellbeing in All Policies’) is a key strategic response to the realisation that policies in sectors where health is not the primary concern may be among the most potent drivers of health improvements ^4– 6^. However, the current evidence base on (cost-) effective SDoH-policies is not well-aligned with public health decision-making processes as it lacks robust quantitative evaluations and appraisals of the likely health effects of available policy options and their prioritisation ^7, 8^. This is compounded by healthy public policy being a complex systems problem that involves: (a) many interdependent causal mechanisms, where effect directions, sizes and timings are not well-understood or captured by dominant research methods; (b) actors with diverse values for whom health and wellbeing may just one of many priorities or no priority at all; and (c) policy design and implementation under conditions of deep uncertainty due to evolving geo-political and economic contexts ^9^.

### Complex systems modelling in policy analysis

Complex systems modelling (CSM) may present a useful way forward. CSM is highly effective for understanding and forecasting the response of physical, natural and social systems to policy change and providing evidence for decision-making under conditions of uncertainty ^9, 10^. CSM can capture hypothesised causal relationships between a multiplicity of diverse factors, using simulation techniques to examine the system-wide consequences of changes made in one or more areas within the system ^11^. CSM offers opportunities for ‘what-if’ simulations, which help identify policy options that are desirable (e.g. cost-effective, high-leverage, acceptable to the public and politicians) and robust (i.e. performing well across a wide range of possible, but uncertain, futures) ^9, 12^. SIPHER will use two different methods: policy-focused microsimulations (individual-level models), which are widely used in parts of UK central government, e.g. the Department for Work and Pensions ^13, 14^ and system dynamics (population-level) models, which have also been used successfully in policy contexts (e.g. housing and energy ^15^). In public health, CSM approaches have had notable impact on infectious disease policy such as for poliomyelitis ^16^ and measles ^17^. Yet, despite a substantial body of literature that describes the systems affecting NCDs and highlights the policy potential of CSM ^18^, examples of CSM influences on NCD policy remain rare ^19, 20^. Exceptions are an agent-based model that shaped tobacco regulation ^21^ and a co-produced system dynamics model that influenced county-level cardiovascular disease policies ^22^. A recent review on CSM in mental health research concludes that participatory or co-production methods which engage with policymakers are beneficial for communication, buy-in and identifying leverage points in the system ^23^.

### Policy partnerships, policy prioritisation and focal outcomes

SIPHER was co-developed with three highly-committed policy partners who represent local, regional and national scales of government:

- Sheffield City Council, a local authority (LA) serving 0.5m people;
- Greater Manchester Combined Authority, a devolved city region of 10 local authorities with 2.8 million people; and
- Scottish Government, representing a devolved nation of 5.4 million people.

Exploiting the opportunities offered by new UK localism and devolution settlements, each partner has put in place ambitious plans to develop new working practices that break down departmental silos and align strategies across policy sectors see 24– 27 to promote wellbeing, prosperity and tackle above-UK average levels of NCDs and inequalities.

Each policy partner prioritised key policy areas for SIPHER to focus on, accounting for the anticipated scale of health and equity impacts deemed achievable, the buy-in across relevant sectors, and the anticipated utility of SIPHER’s models and outputs. Academic and policy partners then jointly selected four ‘whole-systems’ policy areas that exhibited major aspects of complexity – one shared by all policy partners, and one unique to each (see Box 1).

Box 1. **SIPHER’s four initial policy priority areas**

**Inclusive economic growth** (shared). The interplay between economic conditions and health is well recognised ^28^ but to reduce health inequalities, the dividends of increased prosperity need to be shared fairly across society. All partners stressed a need to rebalance their economies (e.g. via poverty reduction, spatial redistribution and inclusive labour markets) to create a virtuous circle of economic, equity and NCD benefits.

**Adverse childhood experiences** (Sheffield). There is strong evidence that stressful and traumatic childhood events (‘ACEs’) lead to worse NCD, inequalities and social outcomes (e.g. crime, poor relationships) ^29^. Sheffield policy partners seek ways to reduce the long-term negative consequences of ACEs.

**Housing** (Greater Manchester). Housing quality impacts on health in multiple ways, including via damp, overcrowding, insecurity-stress and depression, respiratory-related NCD and inequalities ^30^. The focus in Greater Manchester is on interventions to improve the accessibility and affordability of decent housing for all Greater Manchester residents.

**Mental wellbeing** (Scotland). In Scotland, poor mental health is strongly socially patterned, associated with multi-morbidity and a 15-year reduction in life expectancy ^31^. The Scottish Government seeks to promote better mental wellbeing via interventions with education, employment, justice and other key service providers.

Reflecting partners’ priorities, SIPHER proposes to focus on increasing healthy life expectancy, reducing multi-morbidity and closing the health gap between advantaged and disadvantaged population groups. SIPHER will also include broader wellbeing outcomes (measured objectively and subjectively) across a number of relevant domains. The choice of wellbeing domains will be informed by systems mapping – a process described later - but aside from health will likely include financial stability, employment, housing conditions and positive social and community relationships. We will value the economic costs and benefits across sectors, enabling cost-effectiveness estimates that use a societal perspective to tie in with policy partners’ dominant approaches e.g. 32.

## Aims, scientific objectives and theory of change

SIPHER aims to generate transformative health and wellbeing gains via: 1) sustainable, cross-sectoral solutions to prevention; 2) greater policy coherence across sectors; and 3) more confident prevention investment by exploiting synergies between the goals of health and other policy sectors and ensuring that non-health policies do not undermine health and wellbeing goals.

Systems science reframes policies and policymaking as active components in a complex, dynamic implementation landscape ^33^. As such, efforts to appraise and evaluate the impacts of policy decisions on NCDs, wellbeing, inequalities and other outcomes must account for complex causal pathways that include a multiplicity of interacting factors, feedback loops (dampening or amplifying effects over time), non-linearity, adaptive processes (system responses that may anticipate and displace effects) and open boundaries (inputs from and outputs to external systems). In response, SIPHER’s approach blends a thorough understanding of the four policy systems (e.g. decision processes, policymaker beliefs about cause-and-effect, societal values relating to competing policy outcomes) with complex systems policy appraisal. Our multidisciplinary team will harness methodological advances in evidence synthesis, data science, microsimulation, system dynamics modelling, multi-criteria economic evaluation and knowledge mobilisation. There are five key research gaps that SIPHER seeks to address: 1) use of CSM to analyse SDoH and their associated systems and identify leverage points for policy action ^19^; 2) estimation of economic and health economic outcomes of policies in a CSM framework that accounts for system-wide costs and benefits ^34, 35^; 3) inclusion of policy maker understandings of SDoH systems in CSM through sustained co-produced policy analysis and modelling with policy actors; 4) monitoring of SDoH systems using data feeds that provide updating information on system status and projections of policy outcomes, thereby supporting intelligence-led policy refinement; and 5) generation of new understanding of the use of CSM evidence in policy processes.

Our scientific objectives are to co-produce new economic evaluation methods and decision support tools for policymakers which visualise and interactively explore complex systems modelling outputs to inform the design, implementation and ongoing intelligence-led refinement of health and wellbeing-generating policies.

### Research questions

Three overarching research questions will guide the consortium:

1.   How can we capitalise on recent advances in complex systems science and multi-criteria optimisation to maximise the health-generating potential of public policy?

2.   How can we design complex systems research processes, models and decision tools to be most useful to academic and policy audiences?

3.   Which pathways and strategies best translate complex systems science evidence into policy?

### Theory of change

**Figure 1.**
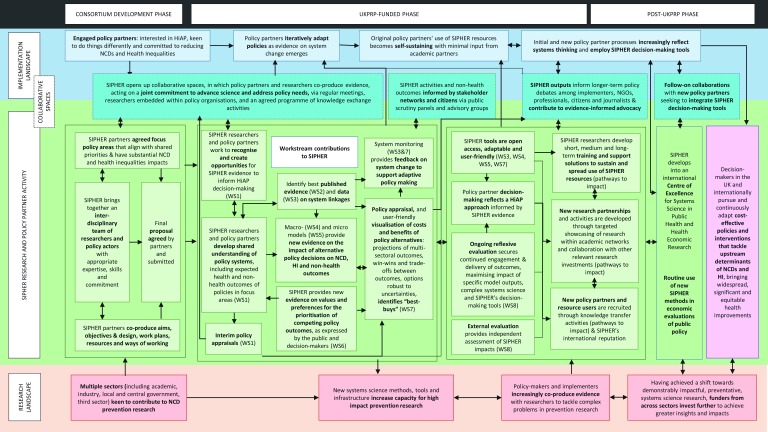
Theory of change.

Figure 1 presents SIPHER’s theory of change, which is mapped onto the UK Prevention Research Partnership Impact and Evaluation Framework. Working with our policy partners, we anticipate our new systems science evidence will initially contribute to policy debates, narratives and agenda-setting in our focus policy areas. As policy opportunities arise, models and tools are then available to answer budget prioritisation and policy design and implementation questions. Specifically, SIPHER will identify which policies maximise benefits across organisational aims, and which policies might lead to important disbenefits in specific sectors. It will also provide ongoing monitoring of policy contexts and effects over time to support policy refinement. In our policy partners’ words, SIPHER evidence will “provide decision makers with the confidence to invest in new approaches”. It is through such evidence-informed investment in SDoH-relevant policies that SIPHER’s NCD and health inequality benefits will be realised. Wider policy networks also benefit: previous research shows that decision models, co-produced with policy-makers to tackle key policy questions, can play a powerful role in advocacy and political process ^36^. Drawing on our experience of policy appraisals and known enablers of evidence use in policy ^37^, we specified the necessary pre-conditions and actions required by academics and partners and created conditions known to facilitate evidence uptake: 1) we have built strong relationships with champions in each organisation which allow us to identify key features in policy debate and ensure our evidence can speak to these; 2) we focus on partners’ policy priorities, and make provision for these to change over time; 3) policy partners are co-investing time and money and will co-own outputs; and 4) we have well-developed plans for the migration of models and decision tools to policy partners via embedded, co-funded analysts. To scale-up our impact, we will: 1) work with knowledge transfer partners to transform decision support practices to routinely consider whether a policy problem would benefit from a systems science approach; and 2) ensure our models and tools transfer between policies and jurisdictions, in recognition that policy makers prefer context-specific results.

## SIPHER workstreams

**Figure 2.**
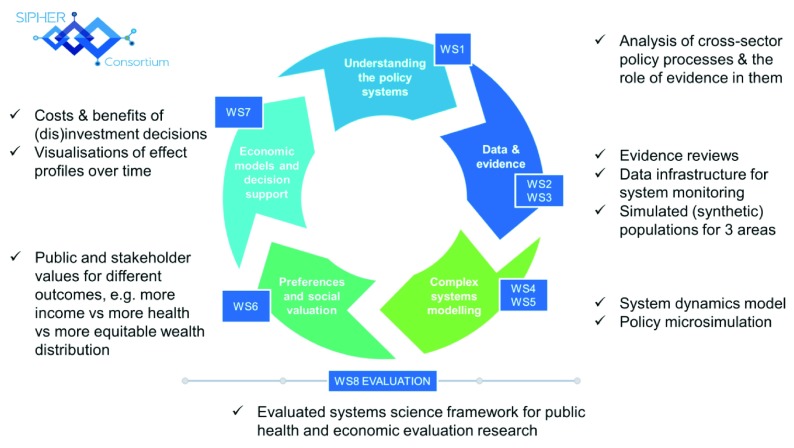
SIPHER workstreams.

SIPHER consists of eight tightly-interwoven workstreams (WS), using a mix of qualitative and quantitative systems science (see Figure 2).

### WS1: Understanding policy systems, policy processes and evidence needs

In WS1, we will formally engage with and capture policy developments in each organisation, including external influences on the organisations’ policy environments. Research in this WS will provide evidence on decision contexts, structures, practices, influencers and engagement with both the SIPHER programme and its outputs. Its longitudinal multi-method study of cross-sector policy development across three government levels provides empirical insights to advance theoretical accounts of policy change and evidence use. WS1 will also be a crucial source of information regarding changing policy contexts for the rest of the SIPHER work programme, for example around external events, influential actors and issue framing. WS1 will use a novel combination of qualitative methods including interviews, ethnographic research, documentary analysis, and participatory group model building (system mapping) to understand the evolving policy systems in our partner sites – the first study of its kind to use this range of methods to collaboratively explore, over time, changing decision contexts, stakeholders’ mental models of the factors influencing each policy system, their attitudes, values, and ways of working.

### WS2: Synthesis of published evidence

WS2 will develop literature search and review strategies that are suitable for supporting complex systems modelling. Rapid evidence reviews will address a range of consortium concerns including the refinement of hypothesised causal links and system maps, population and spatial distribution of policy impact, state transition estimates, preference-based utilities evidence, and cost-benefit evidence. Given the whole-systems scale, the key is the balance between breadth and depth in the review process. We will adopt a dynamic information-seeking approach ^38^ that accommodates ill-defined and fuzzy review questions, diverse evidence formats and addresses the absence of ‘ideal evidence’ for some information needs by making explicit the judgments and assumptions associated with adopting ‘next best’ evidence. We will use recommended rapid, iterative review methods ^39^ to maximise the breadth, efficiency and relevance of the overall review process whilst achieving greater depth where needed (e.g. areas of particular importance or contention to policy partners, evidence to populate models where available data is limited). Expert knowledge will be sought and quantified using formal elicitation frameworks ^40^ where relevant evidence is absent or insufficient.

### WS3: Data acquisition, management and generation of synthetic populations

WS3 will build a secure data infrastructure, create detailed simulated populations and develop a system monitoring function to inform adaptive policymaking. The secure data infrastructure will enable ongoing sharing of sensitive, individual-level data, the creation of detailed simulated (yet representative) populations, and the development of a system monitoring functionality to inform adaptive policymaking. Data acquisition and warehousing draws on existing infrastructure, providing secure data transfer, storage and curation. A comprehensive data audit will identify the type, scale and accessibility of data needed for each focus area. Data requirements will be informed by WS1 system mapping and WS4 and WS5 model requirements. Using microsimulation, we will create attribute-rich population datasets (e.g. disaggregated by age, gender, socioeconomic status and by geography), linked with attributes relevant to the social determinants of health. This will be achieved by combining partner data with established datasets (e.g. census, government surveys). These ‘synthetic’ data preserve the distribution of original data (crucial for modelling inequalities) and combine attributes from multiple sources which would not ordinarily be released together.

### WS4: Causal system dynamics modelling

WS4 will develop novel system dynamics methodology to robustly estimate the health, social and economic impacts of policies. We will develop causal models for inclusive growth, housing, mental wellbeing and ACEs, and parameterise the models for each partner. Causal modelling captures the dynamics and feedback effects of the causal processes represented on WS1 system maps ^41^, for example relationships between unemployment, poverty and healthy life expectancy. The causal relationships between variables on the system maps can be represented at three levels reflecting increasing demands on the supporting evidence base: evidence where we only have effect direction; evidence that provides effect magnitude, and evidence that informs the temporal relationship of cause and effect. Causal models will be built at each of these levels (including re-use of existing models where available ^15, 42^), then multi-level methods will be used, allowing the inputs and outputs of models with different types of evidence to be integrated to give projected outputs at each level ^43^, fully accounting for uncertainty. WS4 will generate open-source tools to accelerate future CSM applications.

### WS5: Policy microsimulation

WS5 will build dynamic microsimulation models for each policy area to produce estimates of distributional (inequality) impacts needed for policy appraisal, along with a quantification of all relevant uncertainties. We will model the impact of changes in relevant parameters (e.g. employment status) on sub-populations (e.g. by sex, age, socioeconomic status) and at a sub-LA spatial resolution and assesses the evolution over time of effects in the system. The outcome is an open-source dynamic microsimulation tool for future applications.

The CSM methods in WS4 and WS5 are complementary: system dynamics models focus on the relationships between higher-level concepts while microsimulation captures the impacts of environment and policy on the characteristics of individuals and households. For example, WS4 might identify that in one of our jurisdictions, increasing the availability of affordable housing would affect disposable income and health. The WS5 micromodel would then assess changes in individual-level housing, income and health to understand distributional impacts. The combined use of micro and macro models will ensure maximum exploitation of evidence from WS2 and WS3 since each requires different types of data.

### WS6: Social valuations of cross-sectoral outcomes and equity implications

WS6 seeks to provide insight into how people value different policy outcomes (e.g. increased income vs health) and different distributions of outcomes (increased total income vs increased income equality), which will allow policy analysts to incorporate information on the relative acceptability of different trade-offs into their policy assessments. In WS6, we will also convert the multiple outcomes that arise from a whole-systems perspective into two common wellbeing measures needed for economic evaluation. Using a combination of online surveys and discussion groups, it estimates statistical relationships between non-health outcomes and a monetary measure of well-being (equivalent income) and an extended quality-adjusted life-years (E-QALY) measure which incorporates non-health outcomes. The resulting information will then be used in WS7 to compare the preference-weighted costs and benefits of action targeted on different policy levers.

### WS7: Economic evaluation and decision support

WS7 will use multi-criteria decision analysis methods to develop a decision support tool with visualisations that allows researchers and partners to interactively explore tensions and synergies across the full range of outcomes (captured by WS3-6) under alternative scenarios. The tool will incorporate values and preferences generated in WS6 and identify options that maximise benefits and minimise opportunity costs across policy outcomes. The economic modelling will also estimate the accrual profile of health, wellbeing and non-health outcomes over time. WS7 draws on distributed, robust multi-objective optimization techniques from engineering ^1, 44^ and methods for adaptive policy design which have yet to be used in public health ^9^. The latter identify strategies that perform well across different plausible future scenarios, updating projections as uncertainties resolve. Decision tools will help visualise and interactively explore conflicts and synergies between outcomes; see how different sets of preferences (stakeholders’ own or society’s) affect best-buy judgments; and see how economic metrics, e.g. net benefit, map to underpinning tangible outcomes. To encourage uptake, tools will be configured to pre-populate relevant analysis templates and integrated into existing and emerging toolkits available to analysts.

### WS8: Evaluation

Guided by our theory of change, WS8 continuously and reflexively evaluates how the consortium’s activities and outputs translate into impact **.** It assesses how the partner organisations engage with the SIPHER programme and the evidence it generates, and evaluates changes in partner organisations’ approach to cross-sector policy decisions. It takes a multi-perspective approach to evaluation: we will conduct a continuous, mixed-methods, reflexive process evaluation of each consortium activity and of our collective contribution, seeking to gauge SIPHER-attributable changes in partner organisations’ and proactively identify and manage challenges. Using baseline and follow-up interviews and the regular researcher, WS and consortium meetings, we will assess the effectiveness of our collaborative working, use of SIPHER outputs, models and decision-support tools in policy conversations and decisions, volume and value of further research commissioned or funded and any evidence of SIPHER’s work gaining traction in policy organisations and academia beyond the initial consortium. Five years from now, we will commission an external evaluation of SIPHER’s influence on health and wellbeing-related policy decisions.

## Public involvement

We will establish meaningful, place-based public involvement within SIPHER to diversify the range of voices heard, and to begin to redress the under-representation of people living in poverty/disadvantaged circumstances in research and debates on inequalities ^45^. We will work closely with relevant community organisations to develop four groups of individuals with lived experience of the selected policy issues. These lived experience scrutiny panels will meet annually to discuss SIPHER’s progress and findings with the SIPHER leadership. Attendees will be encouraged and supported to scrutinise the work by commenting and asking questions in a facilitated discussion. The events will be distinguished from more generic consultation by an assurance that the SIPHER leadership will either commit to acting on or, if necessary, explain why they cannot act on feedback. Attendees will be compensated for their time and expenses, carefully avoiding potential effects on state benefits. With these events as a focal point, we aim to develop ongoing relationships with attendees and relevant community organisations for ongoing dialogue.

## Plans for co-production and knowledge exchange

Co-production with our policy partners underpins SIPHER. During the development of the consortium, academic and policy researchers shared understandings of organisational cultures, policy plans and evidence priorities, agreed SIPHER’s focus policy areas and co-developed the consortium’s workstreams. Box 2 summarises our co-production mechanisms, which create multiple research-policy interfaces. A crucial role is played by full-time embedded SIPHER researchers employed by each policy organisation. These embedded researchers will co-produce all SIPHER models, tools and outputs. They provide insider knowledge on evolving policy priorities and requirements and help transfer skills, models, tools and evidence into the organisations. Together, these mechanisms will enable us to co-create novel responses to contemporary evidence needs by developing CSM and decision support tools that harness the huge wealth of relevant stakeholder know-how, data, tools, models and research evidence.

Box 2. **Summary of co-production mechanisms in our work plans**

- Jointly decide on the focus and direction of SIPHER’s work, including next-step planning (CMG)
- Jointly explore the evolving policy system, evidence needs and policy processes (WS1)
- Co-produce and iteratively refine system maps (WS1)
- Partners provide input to search strategies, evidence selection and identifying grey literature (WS2)
- Partners respond to and shape emerging findings from across SIPHER
- Partners contribute to the design, data collection, analysis and authorship of outputs e.g. briefing documents or academic articles
- Work with partner in-house data teams to identify, extract and/or synthesise appropriate data for models; share skills on data manipulation (WS3, WS4, WS5)
- Via embedded researchers, create data flows allowing partners to monitor system change (WS3)
- Test and validate modelled outputs with policy experts in our partner organisations; applying microsimulation to answer specific in-house research questions (WS5)
- Transfer skills in systems modelling and analysis into partner organisations (WS4, WS5, WS7)
- Development of tool requirements in consultation with policy and knowledge transfer partners (WS7)
- Incorporate the decision tool into routine decision processes, e.g. impact assessments (WS7)

An NIHR Knowledge Mobilisation Research Fellowship focused on maximising the policy utility of SIPHER’s efforts, especially in informing health in all policies and health equity efforts will bring added value. Crucially, SIPHER has the support of a number of knowledge transfer partner organisations, including Public Health England, Public Health Wales, NHS Health Scotland, Local Government Association, Learn Sheffield, Sheffield City Partnership, NICE, the Alan Turing Institute and Edinburgh City Deal, and will seek to link into ongoing work in each organisation. We will work jointly to secure two-way alignment of SIPHER outputs with these organisations’ existing work. For example, we examined Public Health England, National Institute for Health and Care Excellence and Greater Manchester Combined Authority toolkits and developed WS7 to leverage these. We envisage that SIPHER researchers will spend short secondments with KT partners to develop a thorough understanding of ongoing projects and learn how SIPHER outputs can inform or enhance these.

## Opportunities and challenges

We are trialling a new model of working: of policy partners employing research analysts within their organisation, in turn driving new opportunities for true co-produced processes and outcomes that are of relevance to those partners. These opportunities come from being immersed deeply within an organisation’s culture and knowing “how to get things done”, understanding of emerging policy narratives, priorities and framings, accessing data held in the organisation and being able to ensure fit of the decision tools into existing economic evaluation infrastructures and decision processes. Nevertheless, the policy areas SIPHER is concerned with are not necessarily ones that give quick, highly visible “wins”. Therefore, SIPHER will have the greatest impact if there is continued political will to take into account both short-term and long-term consequences of today’s decisions.

We anticipate that the novelty, multidisciplinarity and interdependency of the different aspects of our work will present significant challenges. Finding a common language and being able to align the work of many different teams will require a substantial investment in time, goodwill and careful management ^46^. We fully expect problems of data availability, accessibility and sharing. Finally, the project will need to be mindful of the misalignment between university and partner expectations around what constitutes worthwhile outputs and take care to balance time to plan, produce and disseminate outputs aimed at academic and policy audiences.

## Anticipated outputs and impact

SIPHER is designed to deliver three sets of outputs, as shown in Box 3. If SIPHER is successful in its ambition to give decision-makers the tools and confidence to invest in prevention and consider the wellbeing impacts of policies across all sectors, initial health and wellbeing effects are expected within just a few years. However, most policies tackling social disadvantage and poor living condition have effects that accrue across the lifecourse and may have intergenerational reach. Our theory of change also recognises alternative, less direct, routes in which SIPHER may lead to policy uptake such as informing public attitudes or advocacy action.

Box 3. **SIPHER outputs**

1) A whole-systems economic evaluation methodology for cross-sectoral strategies

- a best-practice evidence review framework for supporting systems modelling
- secure data sharing infrastructure and processes
- digital twins (synthetic populations) for policy simulations in the three partner jurisdictions
- open source models for dynamic simulations adaptable to other contexts/topics
- continuous system monitoring function to support policy refinement
- a co-designed decision support tool that functions at multiple policy levels (local to national)
- guidance and training material

2) Evidence on the effectiveness and cost-effectiveness of policies

- causal mechanisms linking upstream policies with health, wellbeing, economic & inequality outcomes
- influential levers within policy systems
- dispersion and accumulation of effects across population subgroups and policy sectors over time
- synergies and trade-offs between different policy outcomes, and public and stakeholder preferences for trade-offs
- comparative cost and benefit profiles for different (dis)investment options
- policy options that maximise benefits across different sectors’ goals and are acceptable to public and policymakers

3) Translation of systems science evidence into policy action

- new evidence on cross-sector policy processes and the role of data and evidence in these
- evaluated processes for policy actors to engage in complex systems modelling research and
- evaluated processes for scientists to deliver cross-sector policy decision support

The Consortium’s long-term goal, for far-reaching impact, is for our new whole-systems decision support framework to be widely adopted to inform routine considerations of the health and wellbeing impacts of major non-health policies and interventions. We will seek to progressively expand our reach with the help of our knowledge transfer partners and developing academic networks. Therefore, we will evaluate impacts relating to different timescales: 1) the more immediate impacts - on policy narratives, framings and decisions, 2) changes in interim outcomes along the causal pathways, 3) the modelled health, wellbeing and equality effects for major policy developments that SIPHER evidence contributed to, 4) the gradual socialisation of SIPHER approaches into public policy appraisal and evaluation.

## Data availability

No data are associated with this article.
