# Supplementary material for: The SIPHER Consortium: Introducing the new UK hub for systems science in public health and health economic research
Source: Wellcome Open Res. 2019 Nov 12;4:174. [Version 1] doi: 10.12688/wellcomeopenres.15534.1 (PMC6880277; doi:10.12688/wellcomeopenres.15534.1)
Supplement: Peer review history

# Peer review: Reviewer response for version 1

**DOI:** 10.21956/wellcomeopenres.17005.r37014

**Published:** 2019-11-25

**Authors:** Jane West

**Affiliations:** 1 Bradford Institute for Health Research (BIHR), Bradford, UK

This open letter provides a clear and well-described outline of SIPHER, a UK Prevention Research Partnership funded consortium to reduce non-communicable disease risk. SIPHER aims to establish a shift from health policy to healthy public policy by establishing a culture where wellbeing impacts of policies are a core consideration across all areas of government. The consortium includes scientists, and local, regional and national government partners who have shaped SIPHER’s initial policy priority areas of inclusive economic growth, adverse childhood experiences, housing and mental wellbeing. This is a key strength and will help reduce the disconnect between public health and wider public policy. The authors rightly describe healthy public policy as a complex system problem and present a very readable and engaging systems approach to developing transferable models, tools and outputs that can deliver health benefits for the whole population. 

 The scientific quality is excellent and methods are really well-presented despite their complex nature. Co-production and knowledge transfer has been carefully considered and there is a real sense that this work can achieve much needed health considerations across all sectors to drive better policy marked health improvements. I enjoyed reading this open letter and will enjoy following SIPHER’s achievements as the project develops.

I confirm that I have read this submission and believe that I have an appropriate level of expertise to confirm that it is of an acceptable scientific standard.

# Peer review: Reviewer response for version 1

**DOI:** 10.21956/wellcomeopenres.17005.r37016

**Published:** 2019-11-18

**Authors:** Brian Castellani

**Affiliations:** 1 Department of Sociology, Durham University, Durham, UK

The SIPHER Consortium overview is excellent. It is accessible in its review of several otherwise highly complex concepts. The goals are easy to follow (including relevant charts) as well as its pathways to impact, which are both practical and innovative -- the chart is also very well done. All of which leads to their outputs for evaluation, which are likewise novel and cutting-edge, including employing a participatory, co-production approach, thinking about interventions in complex systems and employing complex systems methods for public health. The specific case studies will lead to important, diverse, and context-aware policy insights as well, including how public health prevention sits within a wider policy cycle, economic growth, and the complexities of different places throughout the UK. Finally, SIPHER's new ways of thinking about the complex socio-environmental factors that impact effective prevention will help to advance better end-user useful public health strategies.

I confirm that I have read this submission and believe that I have an appropriate level of expertise to confirm that it is of an acceptable scientific standard.
